# Dynamic spin interchange in a tridentate Fe(iii) Schiff-base compound†

**DOI:** 10.1039/c5sc04577k

**Published:** 2016-03-17

**Authors:** Ana I. Vicente, Abhinav Joseph, Liliana P. Ferreira, Maria de Deus Carvalho, Vítor H. N. Rodrigues, Mathieu Duttine, Hermínio P. Diogo, Manuel E. Minas da Piedade, Maria José Calhorda, Paulo N. Martinho

**Affiliations:** a Centro de Química e Bioquímica, Faculdade de Ciências, Universidade de Lisboa Campo Grande 1749-016 Lisboa Portugal pnmartinho@ciencias.ulisboa.pt; b BioISI, Faculdade de Ciências, Universidade de Lisboa Campo Grande 1749-016 Lisboa Portugal; c Department of Physics, University of Coimbra 3004-516 Coimbra Portugal; d CNRS, Université de Bordeaux, ICMCB 87 avenue du Dr. A. Schweitzer Pessac F-33608 France; e Centro de Química Estrutural, Instituto Superior Técnico, Universidade de Lisboa 1049-001 Lisboa Portugal

## Abstract

The thermosalient effect is still a rare and poorly understood phenomenon, where crystals suddenly jump, bend, twist or explode upon undergoing a thermally activated phase transition. The synthesis and characterisation of the new spin transition Fe(iii) compound [Fe(5-Br-salEen)_2_][ClO_4_] (salEen = *N*-ethyl-*N*-(2-aminoethyl)salicylaldiminate) is described and its thermosalient behaviour reported. It is the first example of a thermosalient effect with a spin transition and magnetic, calorimetric, diffraction, microscopy and computational studies are used to characterise these effects. Both thermosalient effect and spin transition occur around 320 K upon heating and are accompanied by an anisotropic unit cell change with conservation of crystal symmetry that causes a large enough stress of the crystal lattice to induce crystal explosion. This stress can ultimately be traced back to a diffusionless and distortive structural perturbation resulting in a coupled spin transition-thermosalient effect.

## Abstract 2

Dynamic spin interchange where crystals explode with preservation of magnetic memory is observed for a mononuclear hysteretic Fe(iii) Schiff-base compound.

## Graphical abstract

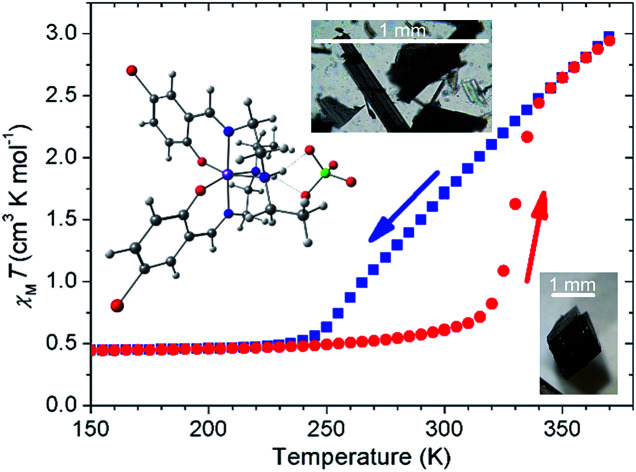

## Introduction

The property of spin interchange displayed by octahedral complexes of d^4^ to d^7^ transition metal ions with application of an external perturbation such as temperature, pressure, magnetic field or light has been extensively studied in the last decade. While the gradual interchange from low-spin (LS) to high-spin (HS) is dubbed spin crossover (SCO), an abrupt conversion between spin states is named spin transition (ST).^1^ In the latter case, transitions are normally associated with a concerted change in ligand conformation and metal–ligand bond length that might sometimes result in efficient communication between metal centres. To be efficient, communication should involve moderate to strong non covalent intermolecular interactions (*e.g.* π–π interactions and H-bonds) which may result in cooperative spin interchange.^2–5^ A strong cooperative effect produces an abrupt change in the magnetic, structural and optical properties of a compound, as the population of both spin states is considerable distinct, sometimes displaying hysteresis. Such a dramatic event at the molecular level may also be accompanied by sudden changes in crystal packing, ultimately inducing cracking or even pulverisation of the crystals.^6^ Often associated with these occurrences, there are changes in the space group that describes the symmetry of the corresponding crystal structure.^7^ That being said, STs take place with crystallographic phase transitions and symmetry breaking.^7^ Most examples of the occurrence of both phenomena have been observed for Fe(ii)^7^ compounds, and only a few for Fe(iii)^8,9^ and Co(ii).^10,11^ Structural phase transitions are a consequence of the concerted movement of ST molecules triggered by either the contraction or expansion of the coordination environment around the metal centre. However, a more intriguing mechanism can be imagined where a crystalline system deprived of an extended network of π–π interactions or H-bonds interactions undergoes a structural phase transition that results in a distortion of the coordination environment around the metal centre and consequently in a change of the energy levels population. This might possibly occur if a compound displays the thermosalient (TS) effect. This effect is driven by a large mechanical response, preservation of crystal symmetry, anisotropic distortion and small structural changes.^12–19^ The crystal retains the same symmetry group with very little overall crystal volume variation and a remarkable mechanical response where crystals either jump, bend, twist or explode. In fact, this effect is distinct from the structural phase transition normally observed in hysteretic ST compounds. In the effect described above, very small overall structural changes and conservation of the crystal symmetry have a large mechanical effect without affecting the magnetic phase of the compound and its paramagnetic behaviour. To date only a very limited number of reports on the TS effect is found in the literature, most of which are organic molecules with one example of an organometallic Pd(ii) compound.^16,18^ Naumov and co-workers^18^ have recently looked into a series of ten compounds that display the thermosalient effect to analyse their structure-kinematic aspects and concluded that while the presence of H-bonds is a key feature for the observation of a TS effect, an extended network of strongly interacting molecules will absorb and channel the release of strain and inhibit the TS effect. In the present work, the TS effect was observed for the spin transition Fe(iii) complex [Fe(5-Br-salEen)_2_]ClO_4_, with the crystals exploding during the LS to HS crossover. Although Weber and co-workers suggest crumbling of the crystals of an iron(ii) compound while changing from HS to LS,^20–23^ their systems possess extended H-bond networks, therefore being very different. Using different characterisation techniques (X-ray crystallography, SQUID magnetometry, differential scanning calorimetry and optical microscopy), it was demonstrated that this effect is accompanied by a ST around room temperature. To the best of our knowledge this is the first reported example of a compound exhibiting a coupled ST–TS effect.

## Experimental

### Materials

*N*-Ethylethylenediamine, 5-bromosalicylaldehyde, sodium perchlorate monohydrate, anhydrous iron(ii) chloride and solvents were purchased and used without further purification in air. IR spectra were recorded on a Perkin Elmer FTIR spectrophotometer. Microanalyses (C, H and N) were measured by the elemental analysis service at the University of Vigo, Spain (**Caution**: Perchlorate salts are notorious for explosiveness; thus precautionary measures must be taken when handling them). 5-Bromosalicylaldehyde (402 mg, 2 mmol) was added to a solution of *N*-ethylethylenediamine (210 μL, 2 mmol) in methanol (30 mL) and left stirring for 15 minutes to give a yellow solution. A solution of iron(ii) chloride (127 mg, 1 mmol) and sodium perchlorate (140 mg, 1 mmol) in methanol (15 mL) was added to the previous reaction mixture and left stirring for 1 h. The purple solution was filtered and deep dark purple cubic crystals were obtained by slow evaporation in an ethanol/acetonitrile 75 : 25 mixture containing [Fe(5-Br-salEen)_2_]ClO_4_ (218 mg, 42%). IR (KBr): *ν*_max_/cm^−1^ 3245 (*ν*_NH_, m), 3074 (*ν*_CH_, w), 1633 (*ν*_C=N_, s), 1591 (*δ*_C=C_, m), 1300 (*ν*_C–N_, s), 1089 (*ν*_ClO_4__, s), 1064 (*ν*_ClO_4__, s), 625 (*ν*_ClO_4__, s). Anal. calcd for C_22_H_28_Br_2_ClFeN_4_O_6_: C, 37.99; H, 4.06; N, 8.05. Found: C, 37.71; H, 4.06; N, 8.16%.

### Physical measurements

Magnetisation measurements as a function of temperature were performed using a SQUID magnetometer (Quantum Design MPMS). The curves were obtained at 1000 Oe for temperatures ranging between 10 and 370 K. Several sequences of cooling and heating were performed using different scan rates (10 K min^−1^, 5 K min^−1^, 2 K min^−1^ and 1 K min^−1^) and measurements were taken in steps of 10 K, 5 K or 2 K, sometimes with one minute waiting after temperature stabilization. Settle mode was used for all temperatures stabilisation.^24^ The collected data were corrected for diamagnetic contributions.

Differential scanning calorimetry (DSC) experiments on [Fe(5-Br-salEen)_2_]ClO_4_ were performed with a temperature-modulated TA Instruments 2920 MTDSC apparatus, operated as a conventional DSC. The liquid nitrogen cooling accessory (LNCA) provided automatic and continuous programmed sample cooling down to 123 K. Heating/cooling rates *β* = 5, 10, and 12 K min^−1^ were used. The sample with a mass *m* = 2.375 mg was sealed under air in an aluminum pan, and weighed to ±0.1 μg on a Mettler UMT2 ultra-micro balance. Helium (Air Liquide N55) at a flow rate of 30 mL min^−1^ was used as the purging gas. Calibration of the temperature scale of the instrument was based on the temperatures of fusion, *T*_fus_, of *n*-decane (*T*_fus_ = 243.75 K), *n*-octadecane (*T*_fus_ = 301.77 K), hexatriacontane (*T*_fus_ = 347.30 K), indium (*T*_fus_ = 430.61 K), and tin (*T*_fus_ = 506.03 K). Onset temperatures were considered. The organic standards were high purity Fluka products and the metal standards were supplied by TA Instruments. The temperature correction for different heating and cooling rates was based on the results obtained for indium. The heat flow scale was calibrated by using indium (Δ_fus_*h* = 28.71 J g^−1^).

Hot-stage microscopy (HSM) was performed with an Olympus BX51 polarizing optical microscope equipped with a Linkam LNP hot-stage and Linkam TMS94 programmable temperature controller. The sample was heated from room temperature to 403 K with a heating rate of 5 K min^−1^ and subsequently cooled at the same rate to 203 K. Images were recorded at selected temperatures using a digital Olympus SC30 camera.

The single crystal X-ray diffraction data were collected with monochromated Mo-Kα radiation (*λ* = 0.71073 Å) on a Bruker SMART Apex II diffractometer equipped a CCD area detector. Data reduction of each compound was carried out using the SAINT-NT software package.^25^ Multi-scan absorption corrections were applied to all raw intensity data using the SADABS program.^26^ The structures were solved by a combination of direct methods with subsequent difference Fourier syntheses and refined by full matrix least squares on *F*^2^ using the SHELX-2014 programs.^27^ The C–H and N–H hydrogen atoms were inserted at geometrical positions with *U*_iso_ proportional to *U*_eq._ of those they are attached. Polymorph i CCDC 1434940; polymorph iii CCDC 1434941. Figures of crystal packing diagrams were drawn with Mercury^28^ and PLATON software package.^29^

Powder X-ray diffraction data were collected with an INEL120 powder diffractometer (Debye–Scherrer geometry; single crystal quartz monochromator and position-sensitive detector, CPS120 by INEL, nominally covering 120° in 2*θ*; wavelength Cu Kalpha1/2: *λ*_1_ = 1.54056 Å and *λ*_2_ = 1.54439 Å; measuring range: 4–115° in 2*θ*). Sample heat bath consisted of a gaseous nitrogen flow from Oxfordcryosystems Helix Controler and Plus Controler, for the single crystal and powder measurements, respectively.

### DFT calculations

Density functional theory (DFT)^30^ calculations were performed with the Gaussian 09 software package^31^ with the hybrid functional PBE1PBE,^32^ and the 6-31G** basis set. Full geometry optimizations without symmetry constraints were carried out for *S* = 1/2 and *S* = 5/2 states. Unrestricted calculations were performed for open shell species. Harmonic vibrational frequencies were calculated for all located stationary structures to confirm that they were minima.

## Results and discussion

**Fig. 1:**
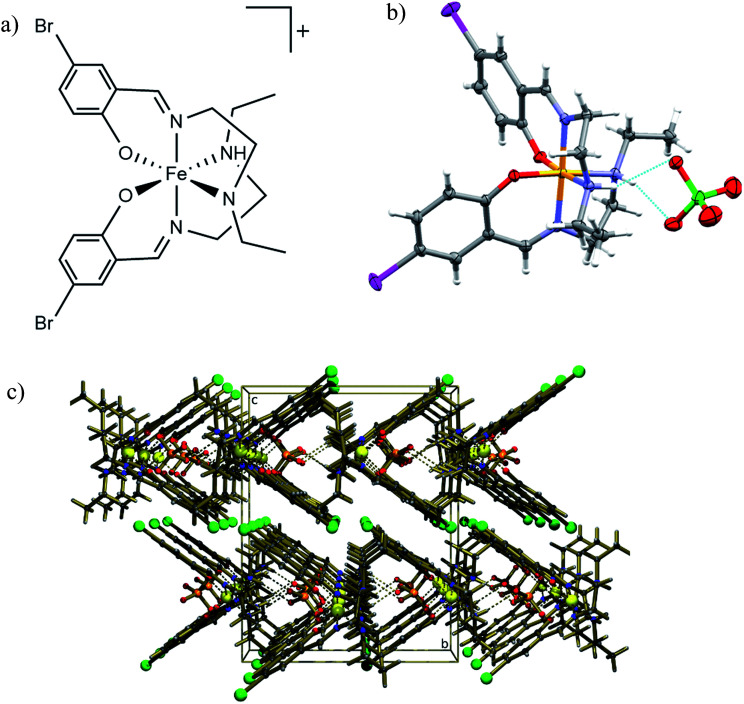
Structural data for [Fe(5-Br-salEen)_2_]ClO_4_ at 125 K. (a) Structure of the complex cation; (b) molecular structure showing the H-bonds between the anion ClO_4_^−^ and the NH groups from the ligands. Grey, light grey, blue, yellow, red, green and pink ellipsoids represent C, H, N, Fe, O, Cl and Br atoms; (c) crystal packing diagram showing rows of complex cations parallel to (100) crystallographic direction forming channels which accommodate the ClO_4_^−^ anions. Gold, light gold, blue, yellow, red, orange and green balls represent C, H, N, Fe, O, Cl and Br atoms.

[Fe(5-Br-salEen)_2_]ClO_4_ was prepared at room temperature from condensation of *N*-ethylethylenediamine (Een) with 5-bromosalicylaldehyde (5-Br-sal), followed by reaction with Fe(ii) chloride and sodium perchlorate, yielding the Fe(iii) crystalline complex, after air oxidation and slow evaporation of the solvent (see methods). IR spectroscopy of a bulk sample showed characteristic stretching bands, at 1633 cm^−1^ (C=N) and 1089 and 1064 cm^−1^ (ClO_4_^−^), and crystals with good quality for single crystal X-ray diffraction were grown by solvent evaporation in air. The structure of the complex was initially determined at 125 K revealing that [Fe(5-Br-salEen)_2_]ClO_4_ crystallises in the orthorhombic space group *Pbcn* with one cation (Fig. 1a) and an ordered perchlorate anion. This is located on a crystallographic 2-fold axis and associated with the cation by two symmetry equivalent N–H⋯O hydrogen bonds (Fig. 1b) with N⋯O distances of 3.061(2) Å and N–H⋯O angles of 156.0(19)°. Fe–O, Fe–N_am_ and Fe–N_im_ bond lengths at this temperature are characteristic of a Fe(iii) centre in the LS state.^33^

The crystal packing view along the *a* crystallographic direction at 125 K emphasises the intercalated rows of cations and anions assembled by N–H⋯O hydrogen bonds running along this direction, as depicted in Fig. 1c. Weak C–H⋯O hydrogen bonds link each anion to the adjacent row. The crystal packing can alternatively be described as consisting of channels of complex cations perpendicular to the (100) crystallographic planes and filled with rows of anions. Additional interactions such as H-bonds, π–π or C–H–π interactions between cations are not observed.

DFT^30^ calculations (GAUSSIAN09,^31^ PBE1PBE^32^) were performed in [Fe(5-Br-salEen)_2_]ClO_4_, based on a realistic model consisting of the cation complex and the corresponding hydrogen bonded perchlorate counter ion, with the same positions found in the crystal structure. The geometries of the HS (*S* = 5/2) and LS (*S* = 1/2) species were fully optimised (see methods) without symmetry constraints. The calculated bond lengths for the LS forms of [Fe(5-Br-salEen)_2_]ClO_4_ agree very well with those experimentally obtained at 125 K (Fig. S1†). These results show that the charge assisted N–H⋯O hydrogen bond directly influences the metal–ligand bonds and, thus, the electronic structure of the Fe(iii) centre. Analogous metal–ligand bonds are significantly longer in the HS isomer, namely the Fe–N_am_ distances increase by at least 0.15 Å and the Fe–N_im_ by almost 0.20 Å. Only the Fe–O bond increases by a much smaller value, 0.05 Å. These calculated distances are much longer than the experimental values determined at RT, suggesting, in agreement with the magnetic data described below, that the metal centre after cooling from 370 K to RT may be far from pure HS. The HS form is the most stable by 31.0 kJ mol^−1^ (electronic energy) and in the absence of the anion by 41.5 kJ mol^−1^, reflecting the anion/cation interaction.

**Fig. 2:**
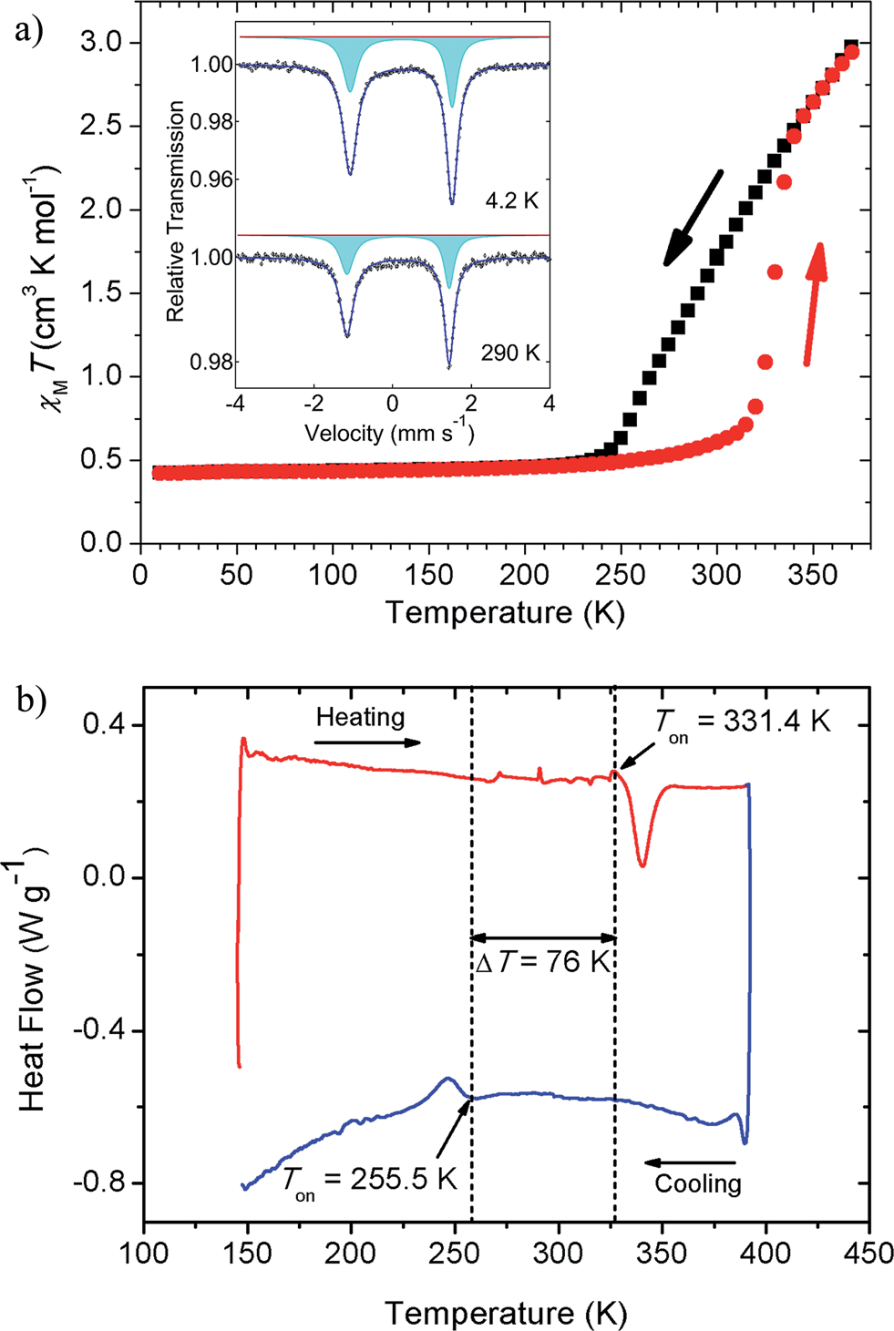
Temperature dependent magnetic measurements and differential scanning calorimetry for [Fe(5-Br-salEen)_2_]ClO_4_. (a) Temperature dependence of *χ*_M_*T*, obtained under 1000 Oe, on cooling (black) and heating (red) modes. Inset: ^57^Fe Mössbauer spectra collected at 4.2 and 290 K; (b) DSC measured curve for a heating/cooling cycle (*m* = 2.375 mg), carried out at *β* = 5 K min^−1^.

The thermal magnetic behaviour of [Fe(5-Br-salEen)_2_]ClO_4_ in the temperature range 10 K to 370 K was investigated by SQUID magnetometry and shows a rather interesting magnetic profile for a mononuclear Fe(iii) ion, with a large hysteresis window at room temperature, Fig. 2. On heating from 10 K, the complex remains in the LS state with a value of *χ*_M_*T* of about 0.40 cm^3^ mol^−1^ K up to approximately 250 K, where the population of the HS state very gradually increases up to 315 K, reaching a *χ*_M_*T* ∼ 0.7 cm^3^ mol^−1^ K. Then *χ*_M_*T* abruptly increases to 2.5 cm^3^ mol^−1^ K over a temperature range of 20 K. The cooling and heating curves diverge between 255 K and 335 K. From the derivative curves of both cooling and heating data an average width of 30 K can be assigned to the hysteresis loop. The magnetic profile of the compound indicates that three processes are involved (one on cooling and two on heating), with the more abrupt heating process suggesting cooperativity. Cooling and heating modes at different scan rates were explored and a study of the kinetic bistability at 300 K was also investigated (see ESI†). After this first cycle, scan rate dependent measurements (eight cycles) were performed at four different rates (10, 5, 2 and 1 K min^−1^) showing only small differences for the fastest scan rates at 10 K step intervals (Fig. S2†). Magnetic relaxation of the system within the thermal hysteresis loop was investigated by kinetic studies at 300 K both in the cooling and heating modes (Fig. S3†). A normal cycle at 2 K min^−1^ with 5 K intervals was run and magnetic measurements at 300 K were performed each 20 s for 15 h, showing no signs of relaxation both in the cooling and heating modes. Therefore, different temperature scan rate measurements and kinetic studies indicate a very stable system within the hysteresis loop over time.

Asymmetries in the magnetic profile are normally associated with solvated samples and the loss of solvent molecules, which is not the case for the present compound. This was demonstrated by thermogravimetric analysis (TGA), which evidenced a mass loss of only 0.56 ± 0.15% with onset at *T*_on_ = 347.3 ± 3.0 K when the sample was heated from room temperature to 410 K at a rate *β* = 5 K min^−1^ (see Fig. S4 and Table S1†). The mass loss is observed more than 10 K above the abrupt transition temperature and the result refers to the mean of five independent determinations. The uncertainty quoted corresponds to twice the standard error of the mean. The magnetic behaviour of the unsolvated [Fe(5-Br-salEen)_2_]ClO_4_ with a 30 K wide thermal hysteresis loop around 300 K is one of the rare examples of mononuclear bistable SCO compounds with large hysteresis windows around room-temperature,^34^ potentially interesting for practical applications in molecular devices.^35^

The general features of the thermal magnetic behaviour of the complex shown in Fig. 2 are also captured by the differential scanning calorimetry (DSC) experiments, in particular the ∼80 K hysteresis window. As illustrated in Fig. 2, on heating the complex from 153 K to 393 K at a rate of 5 K min^−1^, an endothermic peak is detected with onset and maximum temperatures at *T*_on_ = 331.4 K and *T*_max_ = 340.4 K, respectively. This thermal event, with associated enthalpy and entropy changes Δ_trs_*H*^o^_m_ = 7.8 kJ mol^−1^ and Δ_trs_*S*^o^_m_ = 23.5 J K^−1^ mol^−1^, is in close agreement with the abrupt increase of *χ*_M_*T* at 315 K. On cooling the sample from 393 K to 143 K, at the same scan rate, an exothermic peak is observed with *T*_on_ = 255.5 K, *T*_max_ = 246.7 K, Δ_trs_*H*^o^_m_ = 3.6 kJ mol^−1^ and Δ_trs_*S*^o^_m_ = 14.1 J K^−1^ mol^−1^. As shown in Table S2,† the values of *T*_on_, *T*_max_ Δ_trs_*H*^o^_m_ and Δ_trs_*S*^o^_m_ are not significantly affected by subjecting the sample to a sequence of heating and cooling cycles and changing the heating/cooling rate from 5 K min^−1^ to 12 K min^−1^. The difference between the mean values of the onset temperatures of the thermal events detected in heating and cooling modes Δ*T*_on_ = 76 K, perfectly matches the SQUID magnetometry results if the onsets of both the heating and the cooling curves are considered (∼80 K).

^57^Fe Mössbauer spectra were collected at 4.2, 78 and 290 K. The measurements at 290 K were performed before and after heating the sample up to 370 K. All spectra are characterised by a rather asymmetric doublet, inset of Fig. 2a (4.2 and 290 K, before heating). The origin of the asymmetric profile was investigated and is discussed in the ESI (Fig. S5–S8 and Table S3†). The best fits to the experimental data were achieved using a single quadrupole doublet allowing different lines widths, with isomer shift and quadrupole splitting values typical for Fe(iii) ions in the LS state (Table S3†). This assignment is also supported by the low temperature magnetisation results, where *χ*_M_*T* is close to the value expected for the spin only *S* = 1/2, 0.38 cm^3^ mol^−1^ K. The line broadening asymmetry has also been found in other LS Fe(iii) compounds^9,36–38^ and has been attributed to the relatively long paramagnetic relaxation times of the iron when compared to the ^57^Fe nuclear Larmor precession time.

Due to the magnetic hysteresis, different populations of the HS and LS states at 290 K were expected when reaching 290 K from a higher or a lower temperature. Therefore, a Mössbauer spectrum at 290 K after heating the sample up to 370 K was also collected (Fig. S9 and Table S3†) and indicates the coexistence of both HS and LS states, with the predominance of the LS. However, it is not possible to quantify the amount of LS and HS contributions due to a spin flipping rate higher than the frequency associated with the Mössbauer time window (140 ns).

In order to clarify the structural changes with temperature and thus the origin of the hysteretic behaviour of the complex, single crystal X-ray diffraction experiments were carried out in four different thermodynamic states: i (125 K), ii (300 K, after heating from 125 K, 300 K↑), iii (300 K, after cooling from 370 K, 300 K↓) and iv (250 K, after cooling from 370 K, 250 K↓) (Tables S4–S9†). The single crystal measurements revealed structures i, ii and iv to be essentially the same, apart from thermal expansion phenomena and increased thermal vibrations. Nevertheless, a new orthorhombic *Pbcn* structure was found for state iii, corresponding to a different polymorph, that transformed at 250 K into state iv. This indicates that the structure changes differently on heating and cooling, an effect that should be responsible for its hysteretic behaviour (Fig. 2). This new polymorph is characterised by longer *a* and *c* cell parameters and a shorter *b*. Its structure is quite similar to that described above (125 K, Fig. 1). The complex cations hydrogen bonded to perchlorate anions are aligned in rows parallel to *a*, *b* and *c* directions. The cations define large channels running parallel to *a*, where the anions sit. The rigidity of the tridentate ligand coordinated to iron prevents major conformational changes. Only the ethyl groups retain some flexibility, as they are involved in weak interactions. Nonetheless, the change in the cell parameters must certainly correlate with changes in the geometry of the cation–anion units and with their spatial arrangement within the unit cell, however subtle these changes may be. The angle *α* between the two symmetry related aromatic planes of the complex cation, the bond lengths, the hydrogen bonds lengths, and some selected dihedral angles, are the most relevant indicators for a detailed comparison of relevant structural parameters for the four structures and are summarised in Table 1.

**Table 1:** Geometrical parameters of the crystalline structures of [Fe(5-Br-salEen)_2_]ClO_4_ in thermodynamic states i–iv^a^ (distances in Å, angles in °)

	125 K	300 K↑	300 K↓	250 K↓
*a* (Å)	10.3027(4)	10.5910(2)	11.931(3)	10.4363(8)
*b* (Å)	14.2644(6)	14.2497(3)	12.170(3)	14.3026(12)
*c* (Å)	18.1624(7)	18.2420(3)	19.166(5)	18.1740(14)
Fe–O (Å)	1.8684(12)	1.8671(19)	1.868(7)	1.8656(18)
Fe–N_im_ (Å)	1.9361(14)	1.937(2)	2.002(8)	1.934(2)
Fe–N_am_ (Å)	2.0340(14)	2.043(2)	2.103(8)	2.037(2)
N_am_–Cl (Å)	3.7582(16)	3.790(3)	3.748(9)	3.782(3)
N_am_–H⋯O (Å)	3.060(2)	3.105(4)	3.158(13)	3.082(3)
N_am_–H⋯O (°)	157.5(19)	158(3)	149(7)	157(3)
C2–N1–C3–C4 (°)	−173.10(14)	−172.4(3)	−169.5(10)	−172.8(2)
C3–C4–N2–C5 (°)	137.42(16)	136.5(3)	141.1(10)	136.8(3)
C5–C6–C7–O1 (°)	4.7(3)	4.4(4)	−2.7(16)	4.3(4)
*α* (°)	68.474(17)	67.448(30)	73.68(13)	67.736(28)

a The single crystals of [Fe(5-Br-salEen)_2_]ClO_4_ were severely degraded by heating up to temperatures higher than 320 K, states iii and iv were inevitably studied with crystals of poorer quality than states i and ii. Along with the unit cell parameters change, the other most evident difference between the models refined for the crystal structures of states ii and iii is the increased thermal disorder regarding the anion, which is certainly associated with the worsened diffracting quality of the single crystals after heating.

The careful comparison of the values presented in Table 1 allows to conclude that state iii corresponds to a subtly different structure (polymorph) of [Fe(5-Br-salEen)_2_]ClO_4_ whereas states i, ii, and iv are mere temperature driven variations of the same structure. In fact, for state iii the conformation of each 5-Br-salEen around the iron atom is discreetly but significantly different from the one found in the other states. This is reflected in the higher *α* angle (∼74° in iii compared to 67–68° for i, ii, and iv) and similar changes (4–5° in C2–N1–C3–C4 and C3–C4–N2–C5) in the dihedral angles. Fig. S10† illustrates the subtle distinction of ligand conformation in states i and iii.

**Fig. 3:**
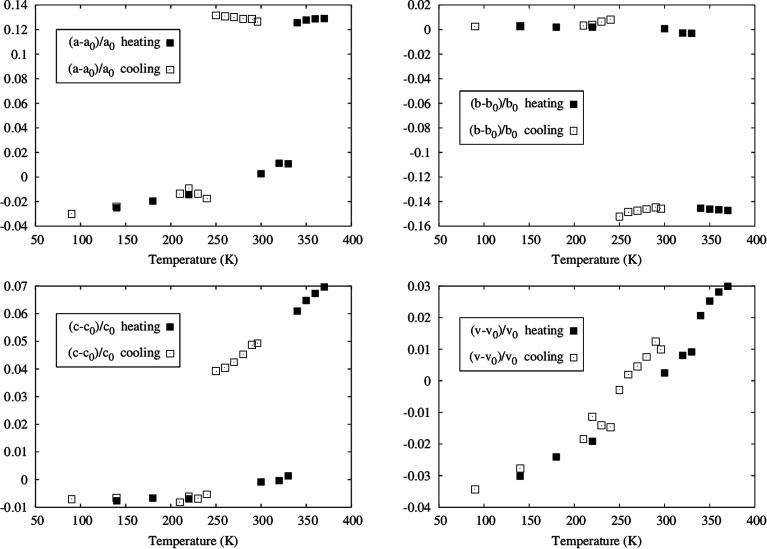
Temperature dependent powder X-ray measurements for [Fe(5-Br-salEen)_2_]ClO_4_. Variation of the cell parameters with temperature as determined from Le Bail refinement of the powder data. The hysteretic behaviour is somewhat attenuated for the volume.

Powder X-ray diffraction measurements were also performed at several temperatures, in order to examine the bulk of the sample and compare with the single crystal data. The data collected at different temperatures, by heating a sample previously cooled to 125 K and by cooling the same sample, after heating it up to 370 K, confirmed the hysteretic behaviour of [Fe(5-Br-salEen)_2_]ClO_4_. Indeed, as shown by the powder patterns in Fig. S11† the difference between the transition temperatures found on heating (between 330 and 340 K) and on cooling (between 240 and 250 K) agrees with those found by DSC and magnetisation measurements. Furthermore, a Le Bail refinement of the powder X-ray data was carried out for each temperature and the resulting cell parameters show also a hysteretic dependence on temperature, as illustrated in Fig. 3. The data obtained from the diffractograms agree with those of the two cells determined with single crystal diffraction for the low and high temperature polymorphs (states i and iii).

The unit cell parameters exhibit an anisotropic change with temperature. In the heating mode, very little variation is observed up to 320 K where two cell parameters dramatically expand (*a* goes from 10.679(2) at 330 K to 11.891(3) at 340 K and *c* goes from 18.293(3) at 330(1) K to 19.382(8) at 340(1) K) and the remaining (*b* goes from 14.206(2) at 330(1) K to 12.179(4) at 340(1) K) dramatically contracts with an overall small cell volume variation.

**Fig. 4:**
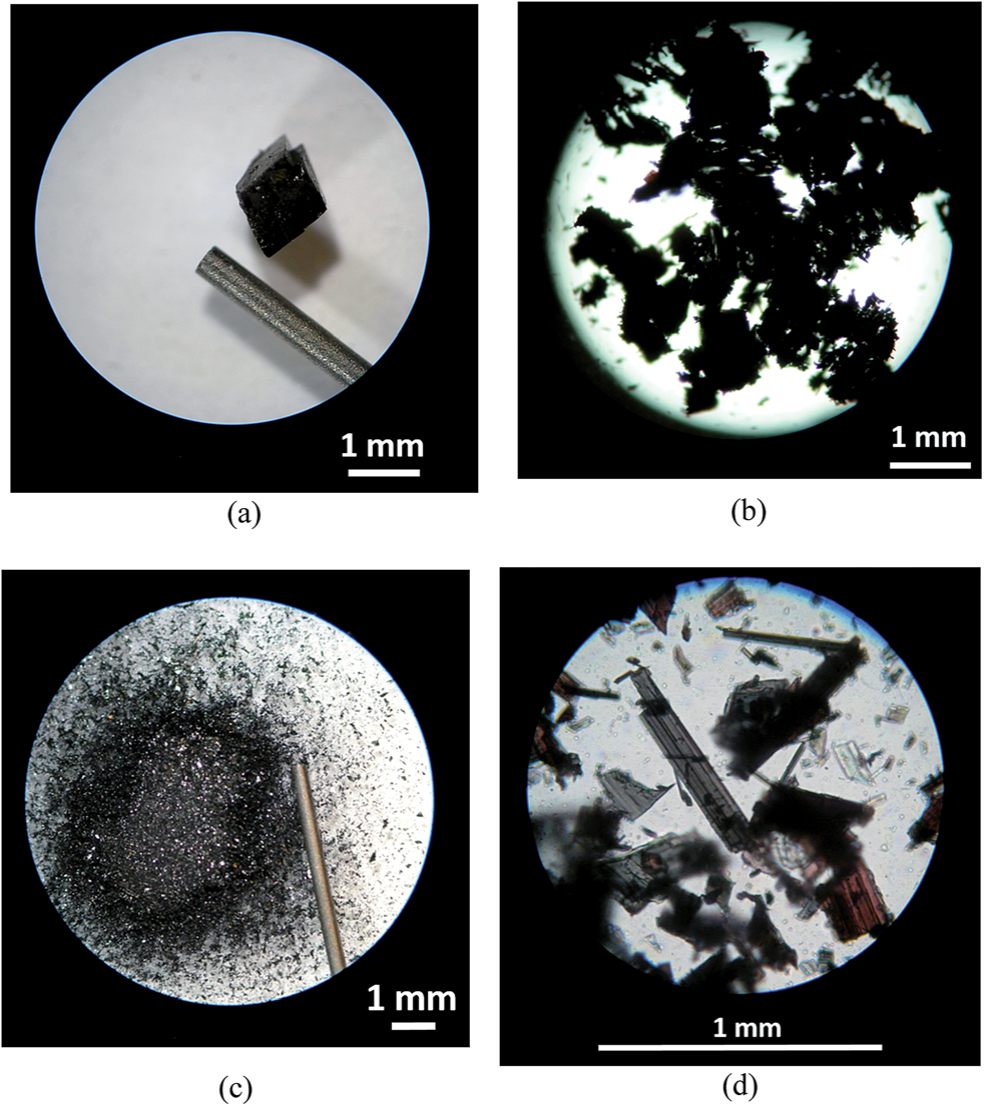
Hot-stage microscopy images illustrating the explosion of a [Fe(5-Br-salEen)_2_]ClO_4_ crystal: (a) crystal at *T* = 320 K (before explosion); (b) fragmented crystal *T* = 337 K (after the first explosion); (c and d) the crystal from (a) after being subjected to four heating–cooling cycles in the range 243–348 K.

Hot-stage microscopy studies on [Fe(5-Br-salEen)_2_]ClO_4_ revealed that the spin transition observed by SQUID magnetometry around 320 K and by DSC at 331.4 K is accompanied by a sudden explosion of the crystals. This is evidenced in Fig. 4, showing images of a [Fe(5-Br-salEen)_2_]ClO_4_ crystal recorded before and after the transition, during four heating–cooling cycles carried out in the range 243–348 K. An average reduction in crystal size of up to 1 : 1000 is estimated after completion of the fourth thermal cycle.

Impressive macroscopic occurrences (crystals jumping, expanding, twisting and/or exploding) together with dramatic unit cell expansion or contraction and very little cell volume variation have been reported before for crystalline compounds exhibiting the thermosalient effect.^12–19^ Typically compounds displaying this effect have in common the preservation of the crystal symmetry, anisotropic distortions, large mechanical responses with small structural changes and a sawtooth DSC profile sensitive to crystal grinding. Naumov and co-workers recently proposed that these compounds can be classified in three different classes according to their molecular shape and potential for intermolecular interactions. For the present compound the thermosalient effect was identified at 320 K by a large mechanical response (crystals explode) with conservation of the crystal symmetry (*Pbcn*), small structural changes (Fig. S10†), anisotropic distortions (while *a* and *c* expand, *b* contracts) and absence of H-bonds extended networks. Although dramatic, these changes do not affect the overall magnetic and thermal behaviour of [Fe(5-Br-salEen)_2_]ClO_4_ over cycles. Together with the TS effect at 320 K, an abrupt change in the magnetic moment is observed suggesting that a cooperative event might also be happening at this temperature. However, extended cooperative networks mediated by either π–π or C–H–π interactions or H-bonds are not observed in the crystal packing of the complex and therefore we can only relate this to the structural changes occurring due to the thermosalient effect. This is therefore the first observation of a coordination compound displaying both the thermosalient effect and a spin transition. Furthermore, both effects do not exist isolated as they are observed at the same temperature (320 K). This can be referred as the first report of a dynamic spin transition effect where the crystals are able to disseminate and retain magnetic memory.

## Conclusions

In summary, the first example of a dynamic spin interchange observed in a perchlorate salt of the Fe(iii) compound [Fe(5-Br-salEen)_2_]^+^ (salEen = *N*-ethyl-*N*-(2-aminoethyl)salicylaldiminate) has been reported. This effect is the result of a concerted mechanism between the thermosalient effect and spin interchange resulting in a structural phase transition and an abrupt spin transition around 320 K. The structural phase transition is the result of a diffusionless and distortive structural perturbation caused by subtle changes in the ligand side chains and the hydrogen bonded perchlorate anions to the cation, inferred from X-ray data and confirmed by DFT calculations. The absence of an extended hydrogen bond network contributes for the building of an internal pressure in the crystals which results in their explosion when the phase transition takes place. It is also interesting to note that this thermosalient effect is only observable in the heating mode, consistent with the increase in thermal energy and resulting in a magnetic hysteresis window of 30 K centred at room temperature. The measurements performed indicate a dynamic system for [Fe(5-Br-salEen)_2_]ClO_4_ where the anisotropic phase transition gives room to accommodate the sudden changes in conformation and bond lengths around the metal centre, resulting in an abrupt spin interchange. This finding opens new opportunities to apply the spin crossover property in dynamic systems.

## Supplementary Material

SC-007-C5SC04577K-s001

SC-007-C5SC04577K-s002

SC-007-C5SC04577K-s003
