# Supplementary material for: Dynamic spin interchange in a tridentate Fe(iii) Schiff-base compound
Source: Chem Sci. 2016 Mar 17;7(7):4251–8. doi: 10.1039/c5sc04577k (PMC6013817; doi:10.1039/c5sc04577k)
Supplement: SC-007-C5SC04577K-s002 [file SC-007-C5SC04577K-s002.pdf]

Electronic Supplementary Material (ESI) for Chemical Science.
This journal is © The Royal Society of Chemistry 2016

SUPPLEMENTARY INFORMATION

Dynamic spin interchange in a tridentate Fe(III) Schiff-base compound

Ana I. Vicente,[a] Abhinav Joseph,[a] Liliana P. Ferreira,[b,c] Maria de Deus Carvalho,[a] Vítor H. N. Rodrigues,[c] Mathieu Duttine,[d] Hermínio P. Diogo,[e] Manuel E. Minas da Piedade,[a] Maria José Calhorda,[a] Paulo Nuno Martinho*[a]

[a] Centro de Química e Bioquímica, Faculdade de Ciências, Universidade de Lisboa, Campo Grande, 1749-016 Lisboa, Portugal. email: pnmartinho@ciencias.ulisboa.pt
[b] BioISI, Faculdade de Ciências, Universidade de Lisboa, Campo Grande, 1749-016 Lisboa, Portugal.
[c] Departamento de Física, Faculdade de Ciências e Tecnologia, Universidade de Coimbra, 3004-516 Coimbra, Portugal.
[d] CNRS, Université de Bordeaux, ICMCB, 87 avenue du Dr. A. Schweitzer, Pessac F-33608, France.
[e] Centro de Química Estrutural, Instituto Superior Técnico, Universidade de Lisboa,1049-001 Lisboa, Portugal.

1. DFT calculations SI2

2. Magnetisation measurements SI3

3. Thermogravimetry experiments SI4

4. Differential scanning calorimetry SI5

5. Mössbauer spectroscopy measurements SI6

6. Structural data SI11

1. DFT calculations

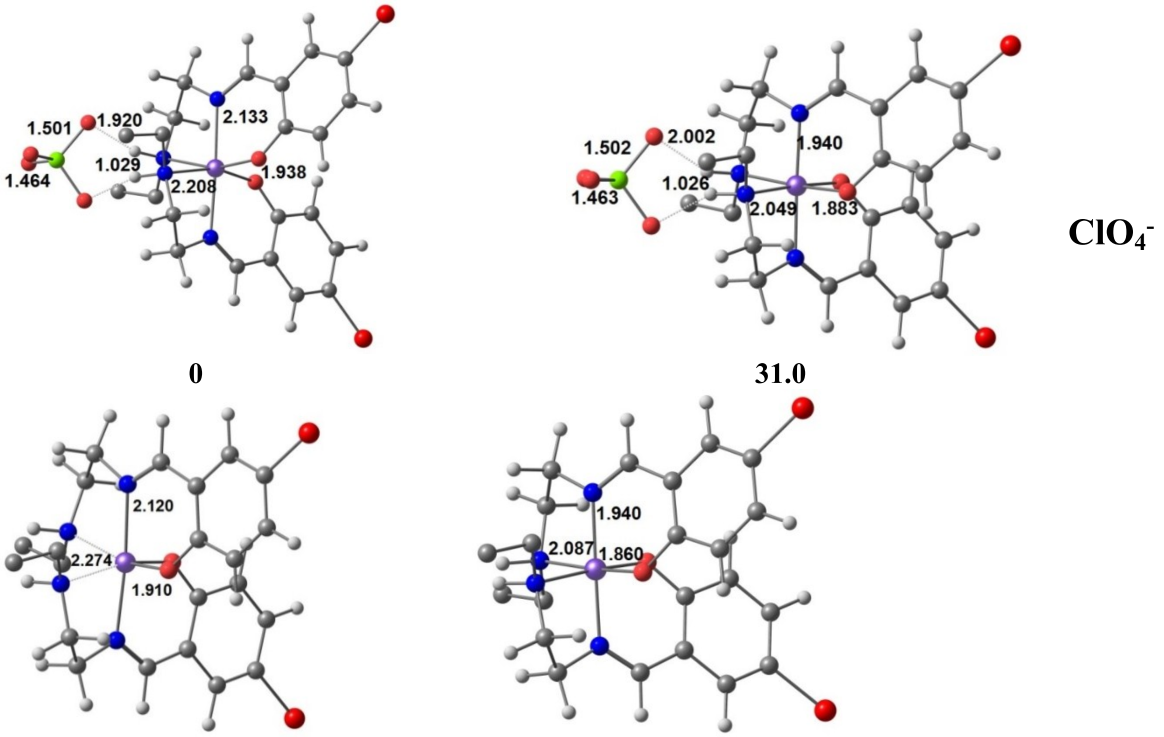

0 **41.5**

HS **LS**

Figure S1 – DFT optimized structures of models [Fe(L)$_2$][ClO$_4^-$] and no anion, for spin states S = 5/2 (left) and S = 1/2 (right), with some relevant distances (Å) and relative energies in kJ mol^{-1}.

2. Magnetisation measurements

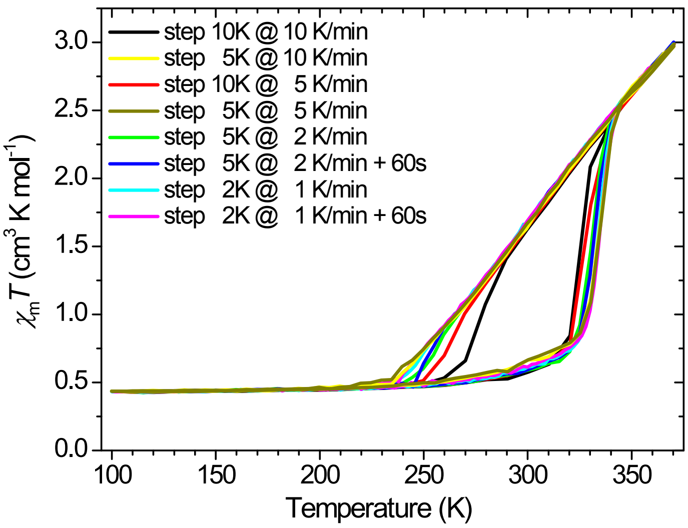

Figure S2 – Scan rate dependent $\chi_m T$ for $[Fe(5\text{-}Br\text{-}salEen)_2]ClO_4$.

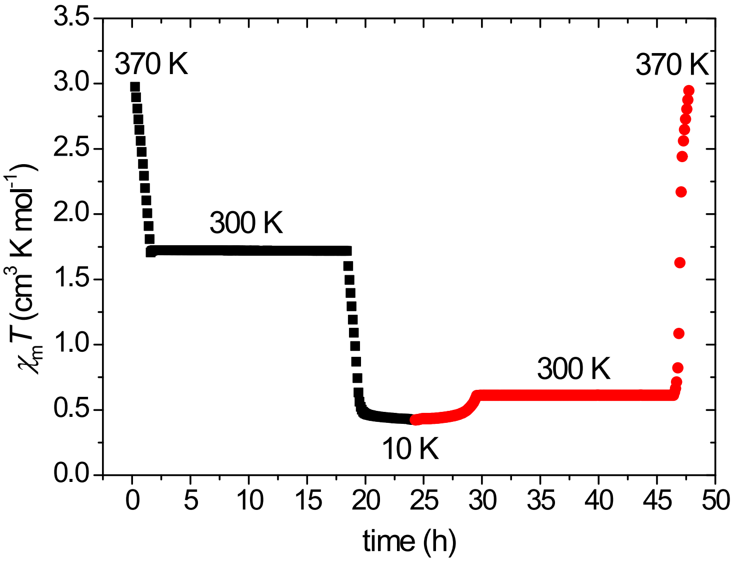

Figure S3 – Time dependent $\chi_m T$ for $[Fe(5\text{-}Br\text{-}salEen)_2]ClO_4$.

3. Thermogravimetry experiments

TGA experiments in the range of 295 K to 425 K were carried out at a heating rate of 5 K min^{-1} on a Perkin Elmer TGA7 apparatus. The balance chamber was kept under a positive flow of nitrogen (Air Liquide N45) of 38 cm^3 min^{-1}. The sample purge gas was also nitrogen (Air Liquide N45) at a flow rate of 22.5 cm^3 min^{-1}. The samples with masses of ~6 mg were placed in an open platinum crucible. The mass scale of the instrument was calibrated with a standard 100 mg weight and the temperature calibration was based on the measurement of the of the Curie points (T_C) of alumel alloy (Perkin-Elmer, T_C = 427.35K) and nickel (Perkin-Elmer, mass fraction 0.9999, T_C = 628.45 K) standard reference materials.

The results of the TG experiments are shown in Figure S4 and Table S1 where m is the initial mass of sample; Δm is the overall mass loss; and T_{on}, T_m, and T_{end} are the temperatures corresponding to the onset, inflection point and end of the mass loss process, respectively. The mean values of the obtained data are: T_{on} =342.7±3.0 K, T_m =342.9±3.0 K, T_{end} =343.7±2.8 K, and Δm = 0.038±0.010 mg. The mean mass loss in percentage is $100\Delta m/m$ = 0.57±0.15%. The uncertainties quoted for all quantities are twice the standard error of the mean.[1]

Table S1 – Results of the thermogravimetry (TGA) experiments on [Fe(5-Br-salEen)$_2$]ClO_4.

m (mg)	T_{on} (K)	T_m (K)	T_{end} (K)	Δm (mg)
6.941	337.82	338.11	339.7	0.022
6.575	347.26	347.65	348.28	0.040
6.700	343.88	343.99	344.43	0.051
6.863	342.25	342.64	342.92	0.035
6.574	342.18	342.23	343.07	0.042

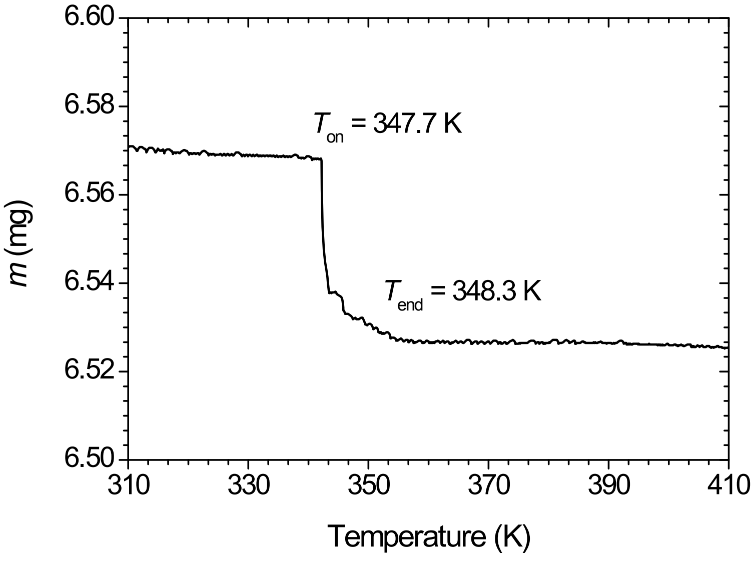

Figure S4 – TGA measuring curve for [Fe(5-Br-salEen)$_2$]ClO_4 (m = 6.575 mg) obtained at a heating rate β = 5 K min^{-1}.

4. Differential scanning calorimetry

Table S2 – Results of the DSC experiments[a]

Run Nr.	β (K min^{-1})	Mode	T_{range} (K)	m (mg)	T_{on} (K)	T_{max} (K)	$\Delta_{trs}H^{o}_{m}$ (kJ mol^{-1})	$\Delta_{trs}S^{o}_{m}$ (J K^{-1} mol^{-1})
1	5	Heating	153-393	2.375	331.42	340.40	7.8	23.54
	5	Cooling	393-143	2.375	255.46	246.68	3.6	14.09
2	5	Heating	153-380	2.375	331.40	339.88	8.3	25.05
	10	Cooling	383-143	2.375	254.49	245.06	3.2	12.57
3	12	Heating	153-393	2.375	330.92	341.03	7.1	21.46

[a]The mass of sample was m = 2.375 mg in all runs and the molar quantities were calculated based on a molar mass $M(C_{22}H_{28}Br_2ClFeN_4O_6)$ = 695.5869 g mol^{-1}.

5. Mössbauer spectroscopy measurements

The Mössbauer spectra were recorded in transmission mode at room temperature and at lower temperatures using a conventional constant-acceleration spectrometer and a 50 mCi ^{57}Co source in a Rh matrix. The low temperature measurements were performed using a liquid helium flow cryostat with a temperature stability of ±0.5 K. The velocity scale was calibrated using an α-Fe foil. The spectra were fitted to Lorentzian lines using the WinNormos software program, and the isomer shifts reported are relative to metallic α-Fe at room temperature.

To understand the origin of the doublet asymmetry, spectra were also recorded in the magic angle configuration (54.7° between the α-ray direction and the normal to the absorber). As can be seen from Figure S5, the spectra collected in the "magic angle" geometry at 4.2 K still exhibits the asymmetric doublet, ruling out the texture effect as a possible cause for the doublet lines asymmetry.[2,3]

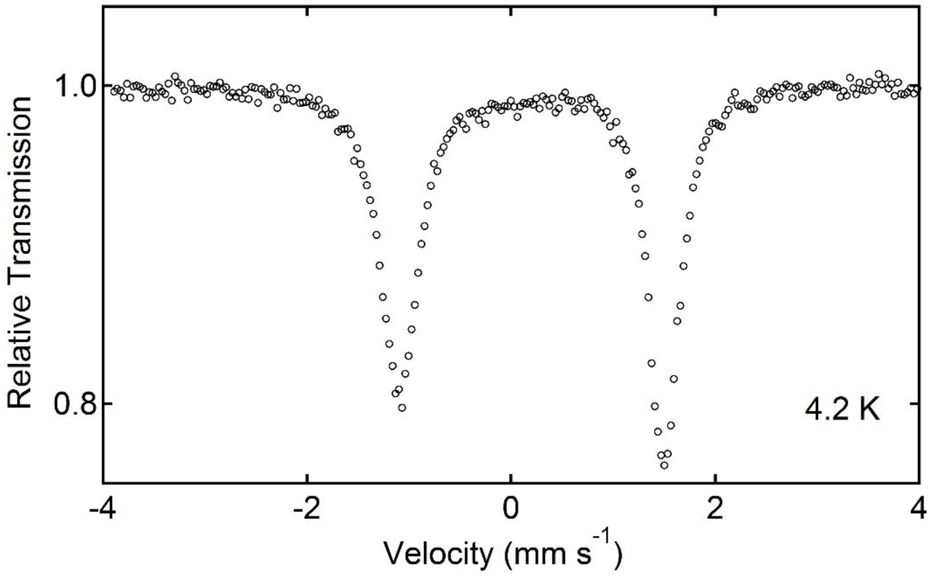

Figure S5 – ^{57}Fe Mössbauer spectrum for $[Fe(5\text{-}Br\text{-}salEen)_2]ClO_4$ at 4.2 K, measured at the "magic angle" (54.7°) in relation to the γ radiation direction.

The spectra measured at different temperatures were also analysed considering two singlet lines. For all temperatures, the same line intensity but different widths were always obtained for the two singlets. Besides, from the guidelines of Figure S6, it is apparent that the asymmetry of the lines is lower at 290 K. These observations led to the conclusion that the Goldankii-Karyagin effect (based on the anisotropy of the Debye-Waller factor for a nucleus in a site with symmetry lower than cubic, predicting different intensities for the doublet lines and that the asymmetry increases with increasing temperature),[4,5] could not explain the lines asymmetry for this complex.

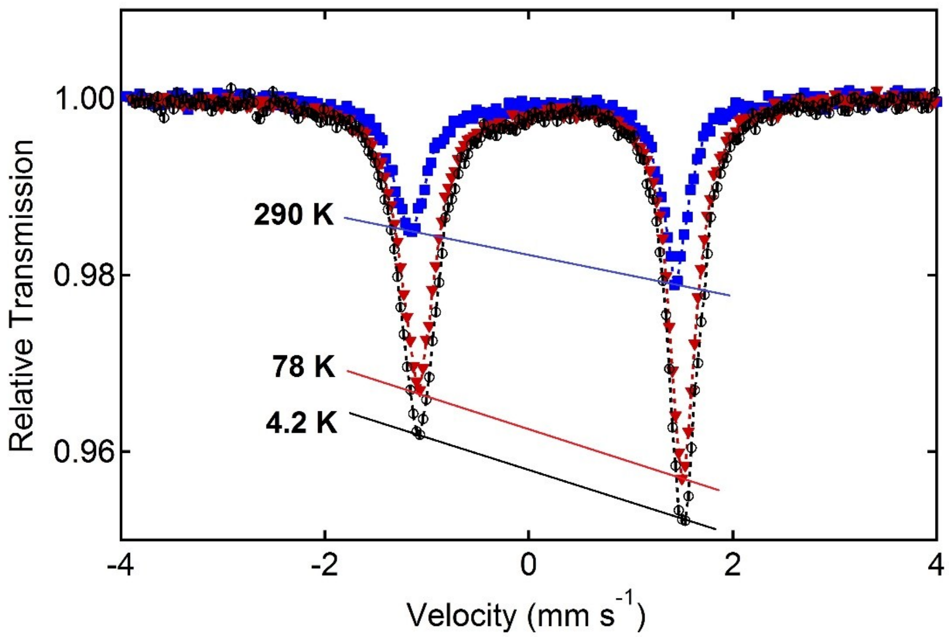

Figure S6 – Superimposed ^{57}Fe Mössbauer spectra for $[Fe(5\text{-}Br\text{-}salEen)_2]ClO_4$ measured at 4.2 K, 78 K and 290 K. The straight lines are guides to the eye, showing that the doublet asymmetry does not increase with temperature.

Figure S7 shows the Mössbauer spectrum at 4.2 K obtained from fittings based on one, two and four quadrupole doublets, assuming equal widths and intensities for both doublet lines. The fits considering either one or two sites did not reproduce correctly the experimental spectrum and the one with four sites gave rise to unreasonably different isomer shift and quadrupole splitting values. Figure S8 displays the same spectrum fitted with one quadrupole doublet, leaving the relative lines width (parameter W_{21}) as an adjustable parameter. Both figures and the fitting parameter values presented in Table S3 show that the best fit is the one displayed in Figure S8, indicating that the quadrupole doublet asymmetry should be related to a relaxation phenomenon. This behavior, found in other LS Fe(III) compounds,[6-9] has been attributed to relatively long paramagnetic relaxation times of the iron when compared to the ^{57}Fe nuclear Larmor precession time.

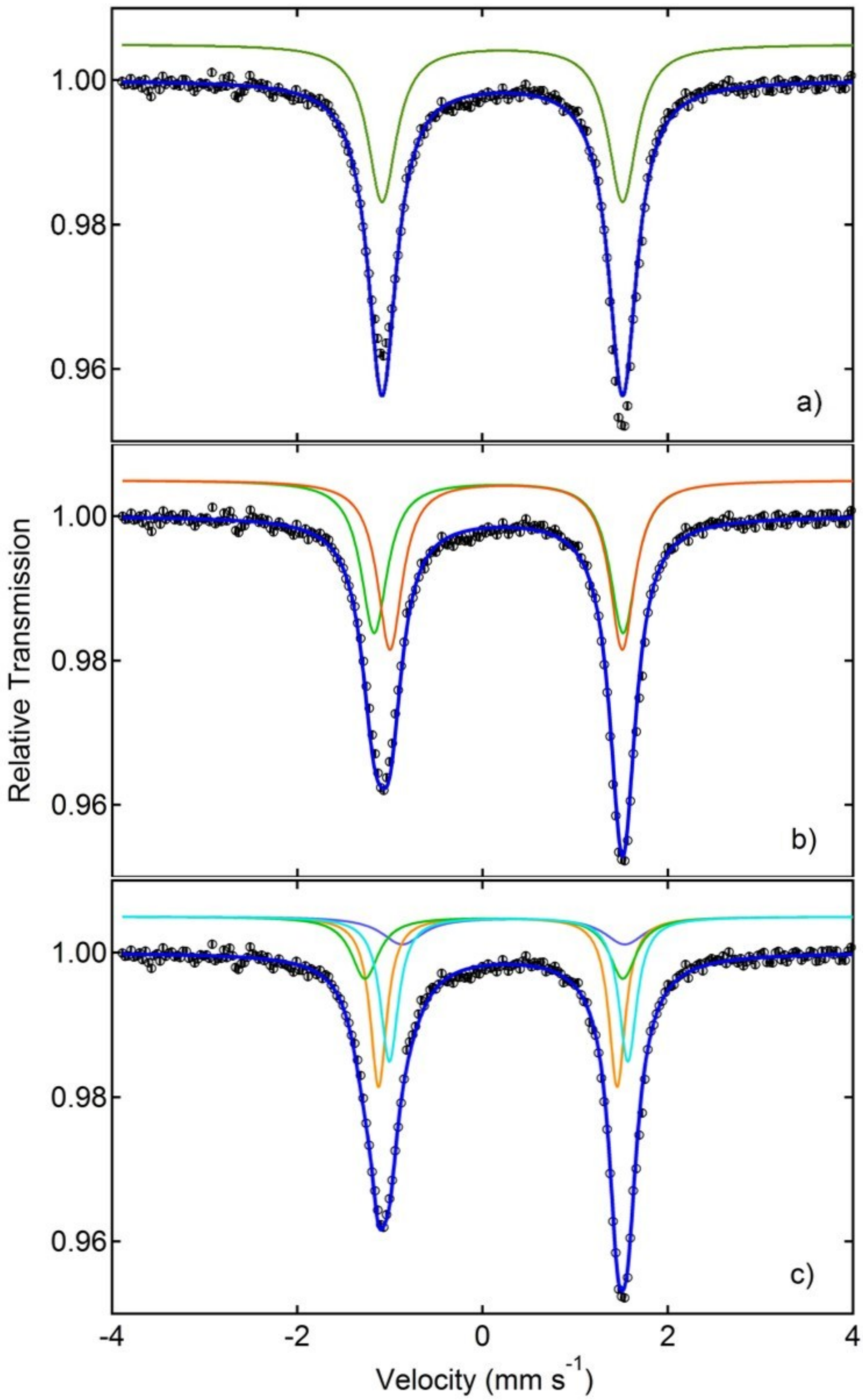

Figure S7 – ^{57}Fe Mössbauer spectrum at 4.2 K fitted by one (a), two (b) and four (c) quadrupole doublets.

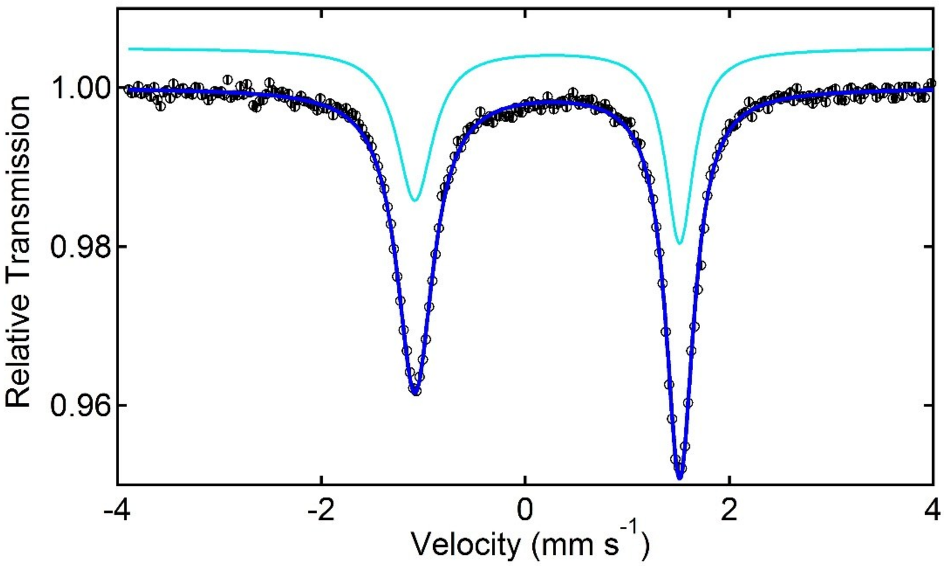

Figure S8 – ^{57}Fe Mössbauer spectrum measured at 4.2 K fitted by one quadrupole doublet leaving the relative line width parameter, W_{21}, as an adjustable parameter.

Table S3 – δ: isomer shift (relative to α-Fe at room temperature); ΔE_Q: quadrupole splitting; Γ: Lorentzian line width at half maximum; W_{21}: width of line 2 relative to line 1; I: relative intensity; χ^2: fitting qui-square. Statistical errors are given in parentheses.

T / K	Site	δ (mm s^{-1})	ΔE_Q (mm s^{-1})	Γ (mm s^{-1})	W_{21} (mm s^{-1})	I (%)	χ^2	Figure
4.2	1	0.216(1)	2.596(2)	0.376(3)	1	100	4.2	S7-a)
	**1**	**0.215(1)**	**2.596(2)**	**0.431(4)**	**0.78(1)**	**100**	**1.2**	**S8**
	1a	0.25(1)	2.51(3)	0.327(6)	1	50	2.2	S7-b)
	2a	0.17(1)	2.69(3)	0.347(7)	1	50		
	1b	0.282(4)	2.57(1)	0.23(1)	1	25	1.5	S7-c)
	2b	0.12(2)	2.79(3)	0.36(2)	1	25		
	3b	0.166(4)	2.58(1)	0.22(7)	1	25		
	4b	0.34(2)	2.40(3)	0.54(3)	1	25		
290 (b.h.)*	1	0.137(2)	2.60(1)	0.41(1)	0.73(1)	100		S9
290 (a.h.)**	1	0.24(1)	2.00(3)	1.79(5)	0.46(1)	100		S9

*b.h. – before heating; **a.h. – after heating the sample up to 370 K.

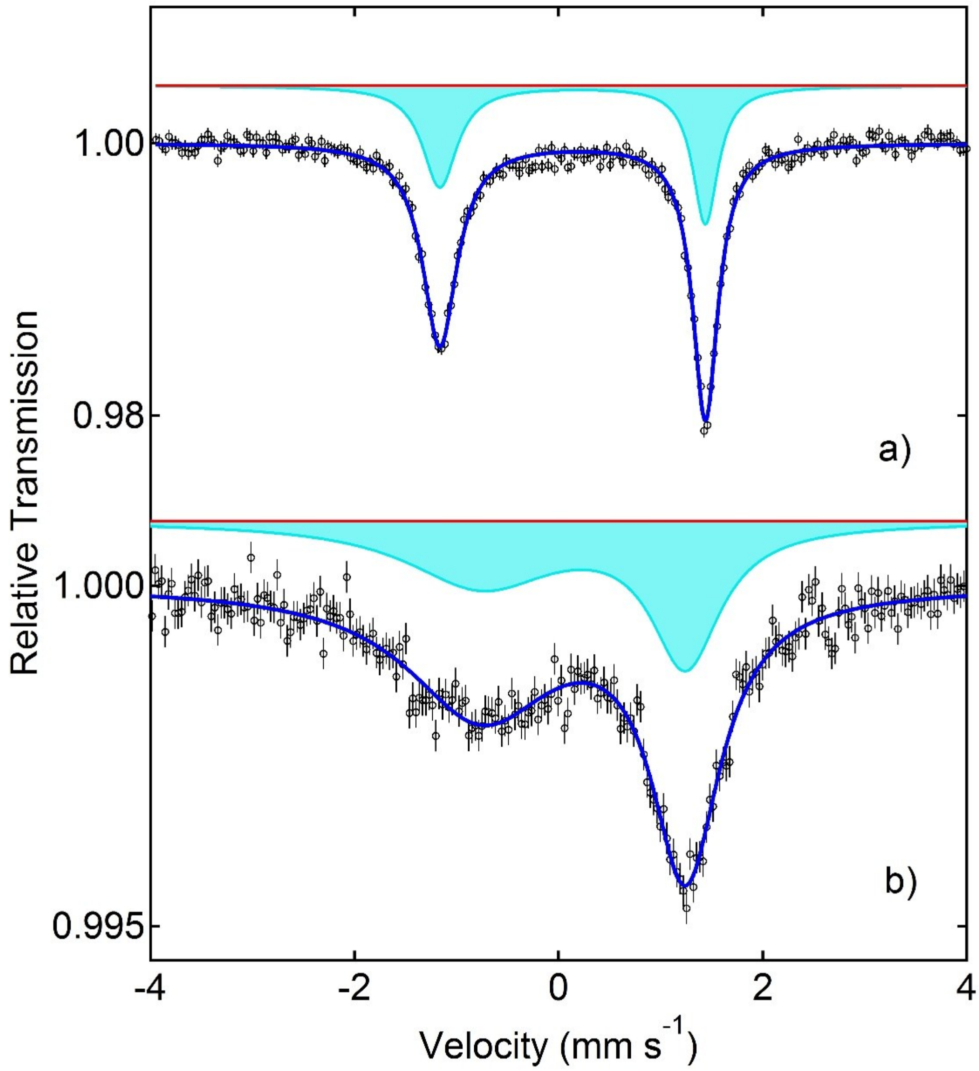

Figure S9 – ^{57}Fe Mössbauer spectra measured at 290 K: a) before heating the sample; b) after heating the sample up to 370 K. The hyperfine fitting parameters are shown in Table S3.

As can be seen through Figure S9 and fitting parameters in Table S3, the 290 K spectrum obtained after heating the sample up to 370 K, although very broad, allows to deduce that the major contribution still comes from LS Fe ions. In fact, the δ value increases from 0.14 mm s^{-1} (at 290 K before heating) to 0.24 mm s^{-1} (at 290 K after heating) indicating a higher contribution of HS states, and the new ΔE_Q value (2.0 mm s^{-1}), although below the one obtained for the 290 K spectrum before heating (2.6 mm s^{-1}) is still well above the ΔE_Q values for HS Fe(III) in similar complexes (< 1 mm s^{-1}, work to be published). Therefore, both hyperfine parameters of the spectrum at 290 K after heating the sample up to 370 K indicate the predominance of LS states and reveal a spin flipping rate higher than $1/\tau_N$, where τ_N is the mean life time of the ^{57}Fe first excited nuclear state (140 ns), not allowing to quantify the amount of LS and HS contributions.

4. Structural data

Table S4 – Crystal data and structure refinement for $[Fe(5\text{-}Br\text{-}salEen)_2]ClO_4$ at 125K↑.

Empirical formula	C22 H28 Br2 Cl Fe N4 O6	
Formula weight	695.60	
Temperature	125(2) K	
Wavelength	0.71073 Å	
Crystal system	Orthorhombic	
Space group	Pbcn	
Unit cell dimensions	a = 10.3027(4) Å	α= 90°.
	b = 14.2644(6) Å	β= 90°.
	c = 18.1624(7) Å	γ = 90°.
Volume	2669.18(18) $Å^3$	
Z	4	
Density (calculated)	1.731 Mg/m^3	
Absorption coefficient	3.703 mm^{-1}	
F(000)	1396	
Crystal size	.12 x .09 x .07 mm^3	
Theta range for data collection	2.243 to 27.494°.	
Index ranges	-13<=h<=13, -14<=k<=18, -23<=l<=23	
Reflections collected	36793	
Independent reflections	3074 [R(int) = 0.0303]	
Completeness to theta = 25.242°	100.0 %	
Absorption correction	Semi-empirical from equivalents	
Max. and min. transmission	1.000000 and 0.772825	
Refinement method	Full-matrix least-squares on F^2	
Data / restraints / parameters	3074 / 0 / 168	
Goodness-of-fit on F^2	1.044	
Final R indices [I>2sigma(I)]	R1 = 0.0231, wR2 = 0.0575	
R indices (all data)	R1 = 0.0309, wR2 = 0.0610	
Extinction coefficient	n/a	
Largest diff. peak and hole	0.575 and -0.424 e.$Å^{-3}$	

Table S5 – Bond lengths [Å] and angles [°] for $[Fe(5\text{-}Br\text{-}salEen)_2]ClO_4$ at 125K↑.

Fe(1)-O(1)#1	1.8684(12)
Fe(1)-O(1)	1.8684(12)
Fe(1)-N(2)#1	1.9361(14)
Fe(1)-N(2)	1.9361(14)
Fe(1)-N(1)#1	2.0340(14)
Fe(1)-N(1)	2.0340(14)
O(1)#1-Fe(1)-O(1)	95.42(7)
O(1)#1-Fe(1)-N(2)#1	92.89(6)
O(1)-Fe(1)-N(2)#1	85.89(6)
O(1)#1-Fe(1)-N(2)	85.89(5)
O(1)-Fe(1)-N(2)	92.89(6)
N(2)#1-Fe(1)-N(2)	178.20(8)
O(1)#1-Fe(1)-N(1)#1	175.48(6)
O(1)-Fe(1)-N(1)#1	87.31(6)
N(2)#1-Fe(1)-N(1)#1	83.70(6)
N(2)-Fe(1)-N(1)#1	97.58(6)
O(1)#1-Fe(1)-N(1)	87.31(6)
O(1)-Fe(1)-N(1)	175.48(6)
N(2)#1-Fe(1)-N(1)	97.58(6)
N(2)-Fe(1)-N(1)	83.70(6)
N(1)#1-Fe(1)-N(1)	90.19(8)

Symmetry transformations used to generate equivalent atoms:
#1 -x+1,y,-z+1/2

Table S6 – Hydrogen bonds for $[Fe(5\text{-}Br\text{-}salEen)_2]ClO_4$ at 125K↑ [Å and °].

D-H...A	d(D-H)	d(H...A)	d(D...A)	<(DHA)
C(2)-H(2A)...N(2)#1	0.99	2.59	3.086(2)	111.1
C(2)-H(2A)...Br(1)#2	0.99	3.08	3.8739(18)	137.7
C(2)-H(2B)...Br(1)#3	0.99	3.10	3.8290(19)	131.2
N(1)-H(1)...Cl(1)	0.84(2)	2.92(2)	3.7582(16)	172.4(18)
N(1)-H(1)...O(2)	0.84(2)	2.27(2)	3.060(2)	157.5(19)
N(1)-H(1)...O(2)#1	0.84(2)	2.60(2)	3.333(2)	146.1(18)
C(3)-H(3B)...Br(1)#3	0.99	3.07	3.8778(18)	139.6
C(5)-H(5)...O(3)#4	0.95	2.57	3.406(3)	146.2

Symmetry transformations used to generate equivalent atoms:
#1 -x+1,y,-z+1/2 #2 x-1/2,y+1/2,-z+1/2 #3 -x+3/2,-y+3/2,z-1/2
#4 x+1/2,y-1/2,-z+1/2

Table S7 – Crystal data and structure refinement for $[Fe(5\text{-}Br\text{-}salEen)_2]ClO_4$ at 300K↓.

Empirical formula	C22 H28 Br2 Cl Fe N4 O6	
Formula weight	695.60	
Temperature	297(2) K	
Wavelength	0.71073 Å	
Crystal system	Orthorhombic	
Space group	Pbcn	
Unit cell dimensions	a = 11.931(3) Å	α= 90°.
	b = 12.170(3) Å	β= 90°.
	c = 19.166(5) Å	γ = 90°.
Volume	2782.9(11) $Å^3$	
Z	4	
Density (calculated)	1.660 Mg/m^3	
Absorption coefficient	3.552 mm^{-1}	
F(000)	1396	
Crystal size	.12 x .09 x .07 mm^3	
Theta range for data collection	2.125 to 24.997°.	
Index ranges	-14<=h<=14, -14<=k<=14, -22<=l<=22	
Reflections collected	29032	
Independent reflections	2454 [R(int) = 0.0404]	
Completeness to theta = 24.997°	100.0 %	
Absorption correction	Semi-empirical from equivalents	
Max. and min. transmission	1.000000 and 0.549286	
Refinement method	Full-matrix least-squares on F^2	
Data / restraints / parameters	2454 / 8 / 150	
Goodness-of-fit on F^2	1.098	
Final R indices [I>2sigma(I)]	R1 = 0.1001, wR2 = 0.2984	
R indices (all data)	R1 = 0.1583, wR2 = 0.3515	
Extinction coefficient	n/a	
Largest diff. peak and hole	1.772 and -2.296 e.$Å^{-3}$	

Table S8 – Bond lengths [Å] and angles [°] for $[Fe(5\text{-}Br\text{-}salEen)_2]ClO_4$ at 300K↓.

Fe(1)-O(1)#1	1.868(7)
Fe(1)-O(1)	1.868(7)
Fe(1)-N(2)	2.002(8)
Fe(1)-N(2)#1	2.002(8)
Fe(1)-N(1)#1	2.103(8)
Fe(1)-N(1)	2.103(8)
O(1)#1-Fe(1)-O(1)	93.8(5)
O(1)#1-Fe(1)-N(2)	91.6(3)
O(1)-Fe(1)-N(2)	92.3(3)
O(1)#1-Fe(1)-N(2)#1	92.3(3)
O(1)-Fe(1)-N(2)#1	91.6(3)
N(2)-Fe(1)-N(2)#1	174.3(5)
O(1)#1-Fe(1)-N(1)#1	173.6(3)
O(1)-Fe(1)-N(1)#1	88.5(4)
N(2)-Fe(1)-N(1)#1	94.3(3)
N(2)#1-Fe(1)-N(1)#1	81.7(3)
O(1)#1-Fe(1)-N(1)	88.5(4)
O(1)-Fe(1)-N(1)	173.6(3)
N(2)-Fe(1)-N(1)	81.7(3)
N(2)#1-Fe(1)-N(1)	94.3(3)
N(1)#1-Fe(1)-N(1)	89.9(5)

Symmetry transformations used to generate equivalent atoms:
#1 -x+1,y,-z+1/2

Table S9 – Hydrogen bonds for $[Fe(5\text{-}Br\text{-}salEen)_2]ClO_4$ at 300K↓ [Å and °].

D-H...A	d(D-H)	d(H...A)	d(D...A)	<(DHA)
C(1)-H(1C)...Br(1)#2	0.96	3.06	3.847(17)	140.1
C(2)-H(2A)...O(1)#1	0.97	2.58	3.063(15)	110.7
C(2)-H(2B)...N(2)#1	0.97	2.59	3.142(14)	115.9
N(1)-H(1)...Cl(1)	0.98	2.76	3.736(11)	174.6
N(1)-H(1)...O(2)	0.98	2.26	3.144(14)	148.8
N(1)-H(1)...O(2)#1	0.98	2.40	3.302(14)	153.5
C(4)-H(4B)...O(2)	0.97	2.56	3.333(16)	137.2
C(5)-H(5)...O(2)#3	0.93	2.54	3.073(12)	117.0

Symmetry transformations used to generate equivalent atoms:
#1 -x+1,y,-z+1/2 #2 -x+3/2,-y+3/2,z-1/2 #3 -x+3/2,y-1/2,z

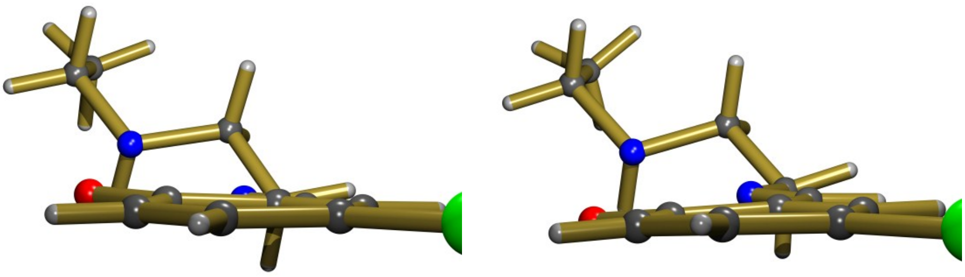

Figure S10 – Strictly equivalent views of the ligand in the complex cation for the state i (125 K), on the left, and state iii (300 K↓), on the right, illustrating conformational differences.

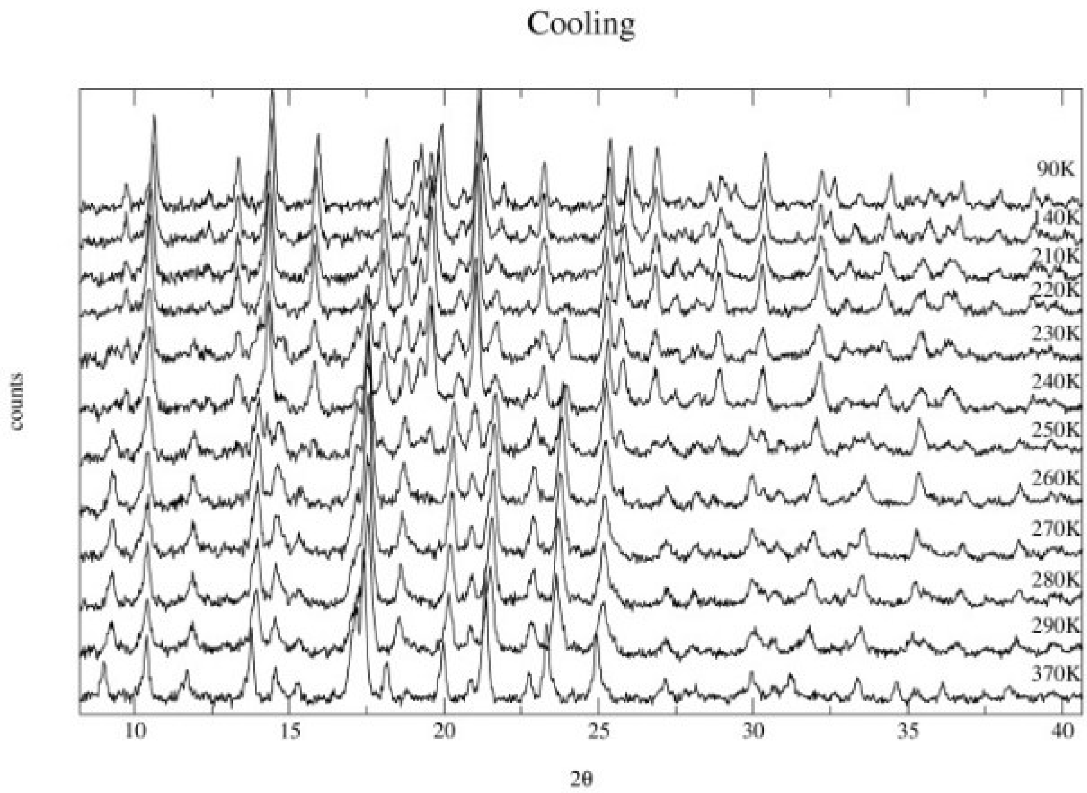

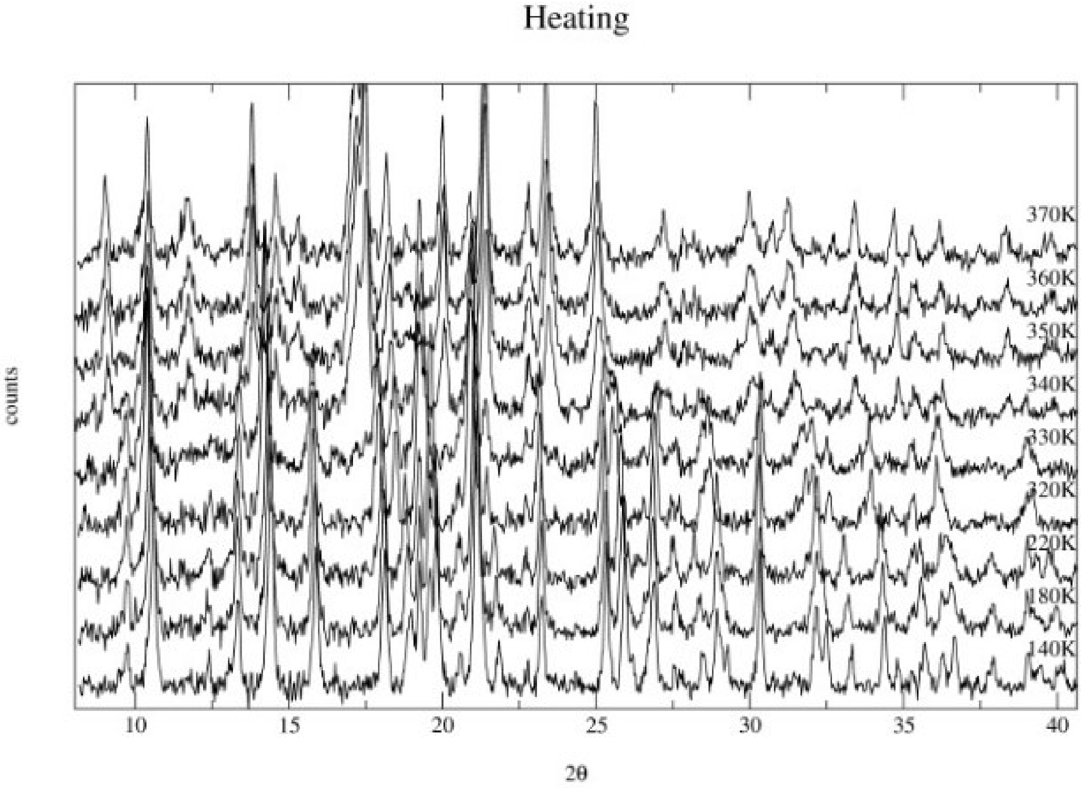

Figure S11 – Powder patterns of [Fe(5-Br-salEen)$_2$]ClO$_4$ at different temperatures. The cooling sequence, starting from 370 K (state iii) is shown at the top; the heating sequence, starting from 140 K (state i) is shown at the bottom.

1. G. Olofsson, *Assignement of Uncertainties; In Experimental Chemical Thermodynamics (Vol. 1)*. Sunner, S.; Mansson, M., Eds., Pergamon Press: Oxford, 1979, 137-159.
2. T. Ericsson and R. Wappling, *J. de Physique*, 1976, **37**, 719–723.
3. J. M. Greneche and F. Varret, *J. Phys. C: Solid State Phys.*, 1982, **15**, 5333-5344.
4. V. I. Goldanskii and R. H. Herber (eds), *Chemical applications of Mössbauer spectroscopy*, Academic Press, 1968, New York-London.
5. H. Spiering, *The electric field gradient and the quadrupole interaction, in Mössbauer spectroscopy applied to inorganic chemistry (Vol. I)*. G.J. Long Ed., Plenum Press, 1984, New York.
6. M. Griffin, S. Shakespeare, H. J. Shepherd, C. J. Harding, J. -F. Létard, C. Desplanches, A. E. Goeta, J. A. K. Howard, A. K. Powell, V. Mereacre, Y. Garcia, A. D. Naik, H. Müller-Bunz and G. G. Morgan, *Angew. Chem. Int. Ed.*, 2011, **50**, 896–900.
7. M. Blume, *Phys. Rev. Lett.*, 1965, **14**, 96–98.
8. P. Gutlich, E. Bill and A. X. Trautwein, *Mössbauer Spectroscopy and Transition Metal Chemistry*, Springer-Verlag, 2011.
9. Y. Maeda, N. Tsutsumi, and Y. Takashima, *Inorg. Chem.*, 1984, **23**, 2440–2447.
